# Gelatin‐starch composite coating containing cucumber peel extract and cumin essential oil: Shelf life improvement of a cheese model

**DOI:** 10.1002/fsn3.2730

**Published:** 2022-01-17

**Authors:** Zahra Esparvarini, Behnaz Bazargani‐Gilani, Mohammadreza Pajohi‐Alamoti, Alireza Nourian

**Affiliations:** ^1^ Department of Food Hygiene and Quality Control Faculty of Veterinary Science Bu‐Ali Sina University Hamedan Iran; ^2^ Department of Pathobiology Faculty of Veterinary Science Bu‐Ali Sina University Hamedan Iran

**Keywords:** antioxidant activity, cucumber peel extract, cumin essential oil, gelatin‐starch coating, shelf life enhancement, UF cheese

## Abstract

In this study, the effects of gelatin‐starch (GS) composite coating containing cucumber peel extract (CPE) and cumin essential oil (CEO) were evaluated on the shelf life enhancement of ultrafiltered (UF) cheese during 56 days of storage under refrigerated conditions. The obtained hydroethanolic CPE by the microwave method showed the best results in terms of the total phenolic content, reducing power, 2,2′‐diphenyl‐1‐picrylhydrazyl (DPPH) activity, and 2,2‐azino‐bis‐3‐ethylbenzothiazoline‐6‐sulfonic acid (ABTS) radical scavenging activity compared to the immersion and ultrasound methods. The studied treatments were as follows: Control (C), GS, CPE, CEO, GS‐CPE, GS‐CEO, and GS‐CPE‐CEO. Scanning electron microscopic surface morphology of treated cheese samples showed the formation of a firm, integrated, flawless, and homogenous layer on the cheese slices of the GS‐CPE‐CEO treatment. All treatments significantly (*p* ≤ .05) decreased the total viable count, psychotropic bacteria, and yeast–mold population compared to the control group. Adding CEO and/or CPE to GS significantly (*p* ≤ .05) controlled undesirable changes in physical characteristics, such as weight, color, and hardness of the cheese slices. Throughout storage time, the coated cheese slices showed more stable chemical features in comparison to the uncoated cheese samples in terms of moisture, lipid oxidation, pH, and titratable acidity (TA). Sensory evaluation of the preparations showed that the GS coating containing CPE and CEO significantly (*p* ≤ .05) had pleasant effects on the sensory features (taste, odor, texture, and overall acceptability) of the cheese samples during storage time. It was concluded that composite coating of GS containing CPE and CEO could improve the microbial, physical, chemical, and sensory features of ultrafiltration (UF) cheese during refrigerated storage.

## Graphical abstract

Gelatin‐starch composite coating containing cucumber peel extract and cumin essential oil could improve the microbial, physical, chemical, and sensory features of ultrafiltered cheese during refrigerated storage.

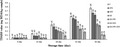

## 1 INTRODUCTION

Ultrafiltration (UF) system concentrates milk fat and protein and is used in cheese manufacture to upgrade cheese yield. The UF cheese is wrapped in plastic packaging in order to prevent environmental contamination and maintain the cheese quality (Fox et al., 2017). Plastics are the most utilized materials in food packaging. Due to the environmental concerns regarding the low recycling rate of plastics, various biodegradable and natural packaging types have been developed to decrease the use of plastic packaging (Cerqueira et al., 2010; Di Pierro et al., 2011). Compared to the plastic, the edible coatings can act as the carriers of active ingredients with antimicrobial and antioxidant properties, which are able to be appropriately distributed in the whole food (Ramos et al., 2012). In addition, the edible coatings produce no waste and residual materials; since, they are biodegradable and easily decomposable in the environment. Some are even consumed with food and can improve the sensory features of it (Cerqueira et al., 2010; Ramos et al., 2012). Carbohydrate polymers are appropriate choices in making coatings and films because of their unique colloidal properties. Starch polysaccharide is an abundant and cheap natural carbohydrate, which can be used in coating production. In order to improve the mechanical properties of starch for coating such as solubility in water and fragility, it can be mixed with a protein polymer, such as gelatin. Gelatin is a collagen‐derived protein that forms a soft, pliable, and elastic gel that can be a good companion to starch rigid gels. Combining hydrophobic substances, such as fatty acids, vegetable oils, resins, surfactants, and waxes in hydrocolloid‐based coatings, is one approach to boost the moisture barrier properties of them. Also, starch‐based films and coatings are considered as selective barriers against oxygen and the incorporation of active agents, such as essential oils (EOs), into the starch coatings can inhibit lipid oxidation, preserving the freshness of product (Fakhouri et al., 2012; Moreno et al., 2018).

The use of EOs in food protection is often limited because of their intense taste, aroma, and potential toxicity. An interesting alternative approach to reduce the release of EOs in food is incorporation of those features into the edible coatings (Ghadermazi et al., 2016). Green cumin (*Cuminium cyminum* L.) is an aromatic plant belonging to the Apiaceae family, which is traditionally used as a condiment in foods. In addition to flavoring and aromatic properties, cumin also has various therapeutic effects (Alizadeh Behbahani et al., 2019, 2020).

Cucumber (*Cucumis sativus*), a member of the Cucurbitaceae family, belongs to traditional Mediterranean diets. Antioxidant and antimicrobial properties of the peel, pulp, and seed extracts of this fruit have been reported in many studies (Fatima et al., 2018; Sotiroudis et al., 2010). Due to the long‐standing interest of Iranians to consume cucumber and cumin seeds along with UF cheese, we decided to evaluate the effects of gelatin‐starch (GS) edible coating containing cucumber peel extract (CPE) and cumin essential oil (CEO) on the physical, chemical, microbial, and sensory characteristics of UF cheese under refrigerated (4 ± 1°C) storage.

## 2 MATERIALS AND METHODS

### 2.1 Extraction of CEO

Fresh cumin seeds were purchased from local markets. CEO was extracted using hydrodistillation method for 3 h by a Clevenger‐type apparatus (Simax, Pyrexfan). Anhydrous sodium sulfate was added to the CEO for dehydration and was preserved in opaque airtight glass vials at 4°C (Alizadeh Behbahani et al., 2020).

### 2.2 Preparation of CPE

Fresh cucumbers were purchased from the local markets, washed, and rinsed by potable water and peeled. Then, cucumber peels were dried in the shade at environment temperature for 2 weeks. The dried samples were ground using a kitchen grinder. The obtained powder was mixed to each of the concentrated ethanol (98%), aqueous ethanol (70%), and water solvents with the ratio of 1:10; then, they were extracted by maceration, ultrasound, and microwave methods. In the maceration procedure, the samples were shaken at 250 rpm (revolutions per minute) for 24 h. Ultrasound apparatus (FAPAN) was used for ultrasound‐assisted extraction (UAE) with the frequency of 20 kHz, the power of 50 W, and the temperature of 25°C for 30 min. In the microwave‐assisted extraction (MAE), the samples were extracted by a microwave oven (SolarDOM, LG) with the power of 360 W for 10 min. Then, the obtained solutions were filtered through filter paper and concentrated by rotary evaporator apparatus (Lab Tech) at 40°C. The remained solvent was removed under vacuum at 50°C. After drying, the extracts were preserved at −18°C until being used (Gallo et al., 2010; Ince et al., 2013).

### 2.3 Antioxidant activity of CPE

#### 2.3.1 Total phenolic content

Total phenolic contents of CPE were measured using the Folin–Ciocalteu reagent assay with gallic acid as a standard. Briefly, 500 μl of the extracts was mixed with 2.25 ml of distilled water (DW) and then, 250 μl of the Folin–Ciocalteu reagent was added. The mixture was vortexed for 1 min and allowed to react for 5 min. Then, 2 ml of sodium carbonate (7.5%) was added. After incubation at room temperature for 120 min, the absorbance of each mixture was measured at 760 nm. The same procedure was also used to a standard solution of gallic acid and a standard curve was prepared. The total phenolic values were considered as mg of gallic acid per gram of the sample (Machu et al., 2015).

#### 2.3.2 Reducing power test

The reducing power of the extracts was measured, according to the modified method of Jemli et al. (2016). One milliliter of extracts was added to 2.5 ml of the sodium phosphate buffer (0.2 M, pH 6.6) and 2.5 ml of potassium ferricyanide (1%). After incubation at 50°C for 20 min, 2.5 ml of trichloroacetic acid (10%) was mixed with the solution and was then centrifuged at 1792 *g* for 10 min. Finally, 2.5 ml of the obtained solution was added to 2.5 ml of the distilled water (DW) and 0.5 ml of ferric chloride (0.1%). After 10 min, the absorbance was read at 700 nm, against blanks that contained all materials except for the samples. Higher absorbance indicated higher reducing power. Butylated hydroxytoluene (BHT) (2 mg/ml) was considered as a positive control.

#### 2.3.3 ABTS radical scavenging activity test

The ABTS radical scavenging activity of the extracts was measured, based on the description of Ozgen et al. (2006). The ABTS (7 mM) and potassium persulfate (2.45 mM) solutions were prepared. They were then mixed together; after 16 h, the obtained solution was diluted with ethanol to earn an absorbance of 0.70 ± 0.02 at 734 nm. Then, 2 ml of the mentioned solution was added to 200 μL of the extract solutions; after incubating for 1 min at room temperature, the absorbance was read at 734 nm using a spectrophotometer (Thermo Spectronic; Helios Gamma). The ABTS radical scavenging activity was calculated as follows:

$$\text{ABTS}\;\text{radical}\;\text{scavenging}\;\text{activity}\;\left(\%\right)=\frac{A_{\text{blank}}‐A_{\text{sample}}}{A_{\text{blank}}}\times 100,$$

where, *A*_blank_ is the absorbance of the blank (containing all materials except for the extract) and *A*_sample_ is the absorbance of the sample.

#### 2.3.4 DPPH free radical scavenging test

The method of Fu et al. (2010) was considered for determining the potency of the samples to scavenge DPPH radical. Fifty microliters of the extracts was mixed to 2 ml of methanol DPPH (24 µg/ml) solution. The obtained solution was stored in dark at environment temperature for 60 min and the absorbance was read at 517 nm, using a spectrophotometer (Thermo Spectronic; Helios Gamma, UK).

$$\text{DPPH}\;\text{radical}\;\text{scavenging}\;\text{activity}\;\left(\%\right)=\frac{A_{\text{blank}}‐A_{\text{sample}}}{A_{\text{blank}}}\times 100,$$

where, *A*_blank_ is the absorbance of the blank (containing all used reagents, except for the sample) and *A*_sample_ is the absorbance of the sample. Butylated hydroxytoluene (BHT) (2 mg/ml) was used as the reference control in all antioxidant activity assays.

### 2.4 Preparation of UF cheese

Fresh UF cheese was purchased from a dairy plant in Hamadan, Iran. Cheeses were made at the Hamadan dairy plant according to the UF cheese‐making method, proposed by the Tetra Pak Company with some modifications by Hesari et al. (2006). After bactofugation, pasteurization (72°C–15 s), ultrafiltration, homogenization, and second pasteurization (80°C–20 s) stages, the retentate with a volume concentration factor of 5.4 kg of milk to 1 kg of retentate entered the starter tank, whereby adding the starter (1 g for 50 kg of retentate), the pH of milk reached the 6.2 level. Then, in the filler, rennet was mixed with water (2 g for 100 kg of retentate) and added to each cheese container. The coagulation tunnel, which was set at 37°C for 30 min, allowed the retentate to be converted into a precheese mixture. In the sealing machine, 4% salt was added onto the parchment paper on the top of cheese; then, the container was sealed using an aluminum foil. In the preripening stage (37°C), after decreasing the cheese pH to 4.80, cheese samples were transferred to a cold room (9 ± 1°C) for cooling and ripening for 3–60 days. Three separate batches following the above procedure were considered for the production of each treatment.

The typical composition of the obtained cheese was as follows: moisture: 60%, fat: 6.75%, protein: 12%, total ash: 6%, pH: 4.35, and TA: 2.25 g of lactic acid/100 g of cheese. Under aseptic conditions, the obtained cheese was divided into seven groups, each containing 110 slices of 10 g and stored at 4°C until being used.

### 2.5 Preparation of coating solutions

Ten grams of gelatin (G) powder was hydrated in 100 ml of distilled water (DW) and agitated by the heater magnetic stirrer (Fan Azma Gostar) in 250 rpm for 1 h under environment temperature. Then, the obtained solution was heated at 70°C for 10 min. Five grams of wheat starch (S) was dissolved in 100 ml of DW and was heated at 70°C for 10 min under constant shaking by the heater magnetic stirrer (Fan Azma Gostar) in 250 rpm. Glycerol (10%) was added as a plasticizer to G and S solutions. In order to obtain the desired edible coating, the resulting solutions of G and S were mixed in the equal ratio of 1:1 by a glass stirrer at environment temperature (Fakhouri et al., 2012).

### 2.6 Preparation of the treatments

**FIGURE 1:**
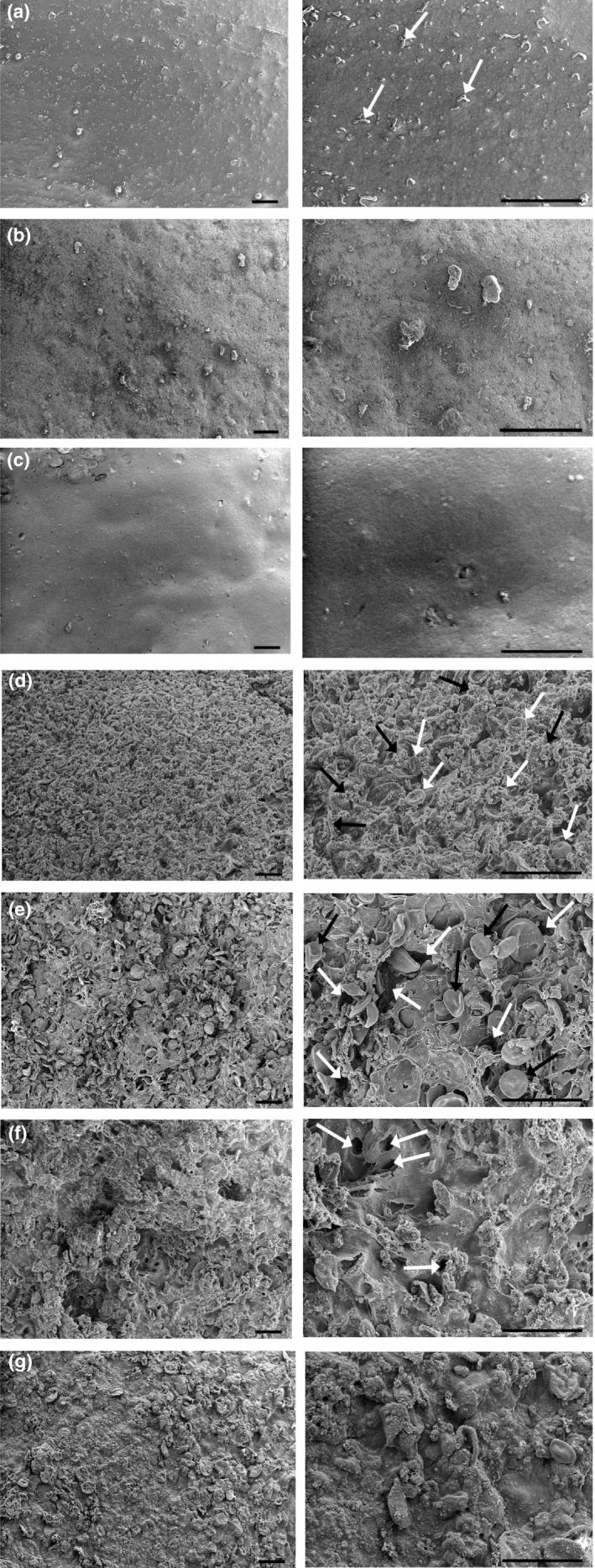
(a–g) View of the treated cheese slices at 0 day of storage. (a) Control (arrows indicate fat globules), (b) CPE, (c) CEO, (d) GS (white and black arrows indicate the starch particles and the gelatin clusters, respectively), (e) GS‐CPE (white and black arrows indicate cavities and starch particles, respectively), (f) GS‐CEO (arrows indicate cavities), and (g) GS‐CPE‐CEO. Treatments: Sterile distilled water (C), cucumber peel extract (CPE), cumin essential oil (CEO), gelatin‐starch edible coating (GS), gelatin‐starch edible coating‐cucumber peel extract (GS‐CPE), gelatin‐starch edible coating‐cumin essential oil (GS‐CEO), and gelatin‐starch edible coating‐cucumber peel extract‐cumin essential oil (GS‐CPE‐CEO). The images on the left and right show magnifications of 100× and 300×, respectively

The final coating solutions consisted of 1‐ Control (C, samples immersed in sterile DW), 2‐ CPE 3%, 3‐ CEO 0.5%, 4‐ GS, 5‐ GS plus CPE 3% (GS‐CPE), 6‐ GS plus CEO 0.5% (GS‐CEO), 7‐ GS plus CPE 3%, and CEO 0.5% (GS‐CPE‐CEO) (Figure 1). Each slice was sunk for 2 min in the respective solutions. Then, the samples were removed and allowed to drain on a sterilized metal net to form the edible coatings; next, the treated samples were packaged in low‐density polyethylene (LDPE) bags.

Finally, all samples were stored at 4°C and analyzed for the microbial, physical, chemical, and sensory features on days 0, 14, 28, 42, and 56 of storage (Ramos et al., 2012).

### 2.7 Scanning electron microscopy (SEM)

A piece of 10 × 10 × 2 mm^3^ from all groups of coated cheeses was cut and immersed overnight in 2.5% buffered glutaraldehyde at 4°C. The samples were then dehydrated in ascending concentrations of ethanol, dried in a critical point drier, and mounted on stubs. The processed samples were sputter‐coated with gold and examined in a scanning electron microscope (JEOL JSM‐840), operating at an accelerating voltage of 10 kV.

### 2.8 Microbiological analysis

In order to measure the microbial population, 10 g of the cheese samples was poured aseptically in a stomacher pouch. After adding 90 ml of 0.1% sterile peptone water (Merck), the mixture was homogenized by a stomacher for 60 s. After serial dilution preparation, the samples were placed in the plates containing plate count agar (PCA) (Merck) and incubated at 35°C for 3 days for the total mesophilic count. Psychotropic bacteria were enumerated on PCA and the plates were incubated at 7°C for 10 days. Rose bengal chloramphenicol (RBC) selective agar (Merck) was used to evaluate the total mold/yeast count after 5 days of incubation at 25°C. Microbiological evaluation was expressed as the log of the number of colony forming units (CFU/g) (Yousef & Carlstrom, 2003).

### 2.9 Weight loss

Weight loss was determined by weighting the samples at the beginning (*W*_1_) and throughout the storage period (*W*_2_). Weight loss of the samples was measured, based on the following equation:

$$\text{Weight}\;\text{loss}\;\left(\%\right)=\frac{W_{1}‐W_{2}}{W_{1}}\times 100,$$

where, *W*_1_ is the weight of the cheese slice in day 0 and *W*_2_ is the weight of the cheese slice in the considered interval (AOAC, 2010).

### 2.10 Hardness

The hardness of the cheese tissues was measured by the penetration test using a digital penetrometer (ZwickRoell, bt1‐fr0 0.5th. d14. Xforce hp) (Pena‐Serna et al., 2016).

### 2.11 Color

Using image processing method, the colors of the cheese slice samples were evaluated (Shahraki et al., 2014). After taking photographs using a single‐lens reflex (SLR) camera (Canon EOS 6D Mark II) in the dark box, the color parameters including L* (brightness/darkness), a* (red/green), and b* (yellow/blue) were determined by Adobe Photoshop Cs5 software (Adobe Systems, Inc.). The overall color difference (Δ*E*) with the control sample was calculated as follows:

$$\Delta E=\sqrt{{\left(L_{s}‐L_{0}\right)}^{2}+{\left(a_{s}‐a_{0}\right)}^{2}+{\left(b_{s}‐b_{0}\right)}^{2}},$$

where, *s* is the color of the sample during the storage period and *L*_0_, *a*_0_, and *b*_0_ are the initial values (1 day after coating application), obtained for cheese under each experimental condition.

### 2.12 Moisture

Moisture content was determined by drying the cheese samples to constant weight at 70°C in a vacuum oven (Fan Azma Gostar).

$$\text{Moisture}\;\text{content}\;\left(\%\right)=\frac{W_{1}‐W_{2}}{W_{1}}\times 100,$$

where, *W*_1_ is the weight of the cheese slice before drying and *W*_2_ is the weight of the cheese slice after drying (AOAC, 2010).

### 2.13 Ash

Total ash content was determined by incineration of the samples at 550°C in an electric furnace (Fan Azma Gostar) (AOAC, 2010). The total ash content of the samples was calculated as follows:

$$\text{Total}\;\text{ash}\;\left(\%\right)=\frac{\text{Weight}\;\text{of}\;\text{obtained}\;\text{ash}}{\text{Weight}\;\text{of}\;\text{the}\;\text{sample}}\times 100.$$

### 2.14 Protein

Protein determination was carried out by the Kjeldahl set (Simax, Pyrexfan). The amount (1 g) of the sample was weighted and digested by concentrated sulfuric acid under heat in a Kjeldahl flask. The obtained solution was then distilled with 50 ml of sodium hydroxide (40%). An Erlenmeyer flask containing 25 ml of boric acid (2%) and protein indicator was determined to obtain the distillate until green discoloration of boric acid. For measuring the total nitrogen index, green discolored boric acid solution was titrated by 0.1 N of the sulfuric acid solution in order to achieve the primary color. The results were mentioned as milligrams of nitrogen per 100 g of the sample. Furthermore, the total protein content was calculated, multiplying the nitrogen content by the conversion factor 6.38 (AOAC, 2010).

### 2.15 Lipid

Fat content of the samples was measured by the Gerber method. Three grams of the sample was added to a butyrometer. After adding 10 ml of sulfuric acid (20%) and 1 ml of amyl alcohol to the butyrometer, it was placed in a hot water bath at 65°C for 5 min. The butyrometer was then centrifuged at 136 *g* for 5 min. Then, the amount of extracted fat in the butyrometer was calculated in percentage (AOAC, 2010).

### 2.16 Titratable acidity

Ten grams of the sample was added to 50 ml of distilled water and homogenized by homogenizer (IKA Ultra‐Turrax T8) for 5 min. The homogenates were heated at 40°C under stirring and were diluted to the final volume of 150 ml with distilled water. After centrifugation at 3000 *g* for 10 min, the supernatants were filtered through paper filter. Then, five drops of phenolphthalein (1% in ethanol) were added to 25 ml of filtered supernatant and the titratable acidity (TA) was determined by the addition of 0.1 N sodium hydroxide until the solution became pink. The TA was calculated as follows:

$$\text{TA}=\frac{a\times b}{c}\times 100,$$

where, *a* and *b* are the concentration and the volume of titrant solution, respectively, and *c* refers to the grams of the analyzed sample (AOAC, 2010).

### 2.17 pH

Ten grams of the sample was added to 50 ml of distilled water and homogenized by a homogenizer (IKA Ultra‐Turrax T8) for 5 min. The homogenates were heated at 40°C under stirring and were diluted to the final volume of 150 ml with distilled water. After centrifugation at 3000 *g* for 10 min, the supernatants were filtered through paper filters. Then, pH measurement was carried out on a filtrate using a pH meter (Jenway) (AOAC, 2010).

### 2.18 Thiobarbituric acid reactive substances

Lipid oxidation of the cheese slices was determined by the thiobarbituric acid reactive substances (TBARS) method, according to Unalan et al. (2013). For analysis, 10 g of the sample was homogenized in a warming blender using a mini jar (Waring Commercial, CAC 134) for 3 min at high speed with 50 ml of TBA (0.38%) and trichloroacetic acid (15%), prepared in HCl solution (0.25 N). Aliquots (5 ml) obtained from the homogenate were incubated in a water bath at 95°C for 15 min for color development. The samples were cooled for 10 min and then centrifuged at 4500 *g* for 25 min. The absorbance of the mixture reaction was measured at the wavelength of 532 nm with a spectrophotometer. A standard curve was generated using 1,1,3,3‐tetraethoxypropane (TEP) and the data were considered as mg of malondialdehyde (MDA) per kg of cheese.

### 2.19 Sensory analysis

A total of 20 undergraduate students (10 females and 10 males, 20–30 years old) of the Food Hygiene and Quality Control Department of BU‐Ali Sina University were chosen as panelists for sensory evaluation of the treatments. A 10 g piece of each cheese sample was tested by the panelists. A 5‐point Hedonic scale was used to evaluate the taste (1: Extremely undesirable, 5: Extremely great), odor (1: Extremely unacceptable/off‐odors, 5: Extremely pleasant), texture (1: Extremely nonpalatable, 5: Extremely palatable), and overall acceptability (1: Extremely unacceptable, 5: Extremely pleasant) (Bazargani‐Gilani & Pajohi‐Alamoti, 2020; Ramos et al., 2012).

### 2.20 Statistical analysis

The obtained data were statistically analyzed by SPSS software (IBM SPSS Statistics V. 21) and reported as mean ± standard deviations (*SD*). The one‐way analysis of variance (ANOVA) and Tukey post hoc test at the significance level of *p* ≤ .05 were used to compare the means. The graphs were prepared by Microsoft Excel 2016 software (Microsoft).

## 3 RESULTS AND DISCUSSION

### 3.1 Antioxidant activity of CPE

**FIGURE 2:**
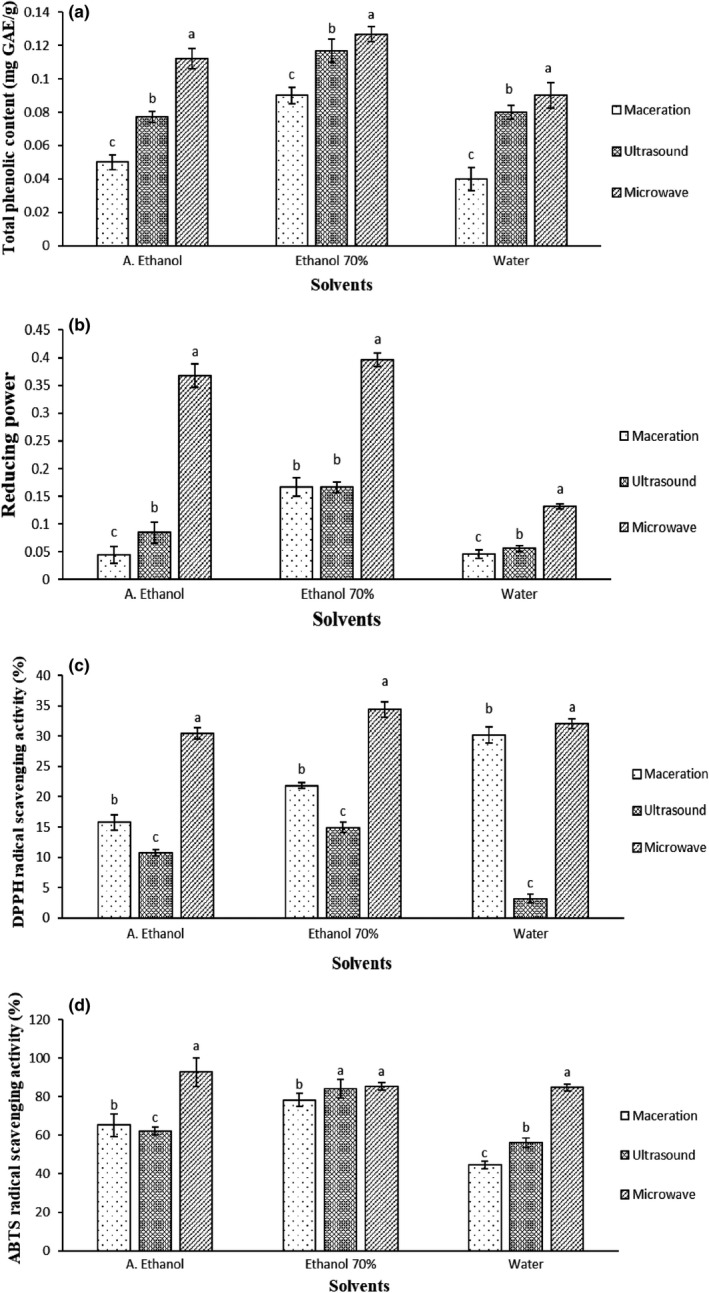
Total phenolic content (a), reducing power (b), 2,2′‐diphenyl‐1‐picrylhydrazyl (DPPH) radical scavenging activity (c), and 2,2‐azino‐bis‐3‐ethylbenzothiazoline‐6‐sulfonic acid (ABTS) radical scavenging activity (d) of the cucumber peel extracts (CPEs) by three extraction methods. Different letters (a, b, c) indicate a statistically significant difference (*p* ≤ .05)

UAE and MAE are new extraction approaches for obtaining antioxidant substances of fruits. ANOVA showed that the extraction method and the used solvent significantly (*p* ≤ .05) affected the total phenolic content and antioxidant activity of CPE. As seen in Figure 2a–d, using MAE with ethanol 70% was the most effective condition in the release of phenolic contents (Figure 2a) and antioxidant activity of CPE (Figure 2b–d) among other conditions. In agreement with our findings, Gallo et al. (2010) reported the higher antioxidant activity of four different medicinal plants (*Cinnamomum zeylanicum*, *Coriandrum sativum*, *Cuminum cyminum*, and *Crocus sativus*) extracts, using MAE compared to the UAE method. This can be associated with the richer content of the antioxidant compounds in the extracts. The authors concluded that the MAE method showed notable superiority in terms of extraction yield and time saving. Ince et al. (2013) suggested the MAE as the most efficient method for the extraction of phenolic compounds of Melissa plant (*Melissa offinalis*). This efficiency can be correlated to the high adsorption of microwave heat by the plant cell water, which leads to increasing water vapor pressure, followed by the destruction of the cell wall and release of the phenolic compounds into the solvent. In addition, MAE is a rapid and environmental‐friendly method, minimizing solvent consumption. The generated heat by the microwave method has a great role in the yield of the phenolic compound extraction. For fast heating, the used solvent must have a high dielectric constant (such as ethanol/water) (Moret et al., 2019). Agarwal et al. (2012) measured the total phenolic content and antioxidant activity of CPE and concluded that this preparation can be introduced as a rich source of antioxidant substances with natural origin. In another study, the total antioxidant activity of various cucumber parts (pulp, peel, and juice) was determined by DPPH radical scavenging activity test and their antioxidant activities were in the following order: pulp > peel > juice. The authors suggested that the antioxidant activity of cucumber extract is due to the presence of a wide range of bioactives, including phenolics, glycosides, oligomer and polymer peptides, organic acids, flavonols, and proanthocyanidins (Sotiroudis et al., 2010). Chen et al. (2019) reported the high radica

l scavenging rate (80%) and low reducing power of cucumber polysaccharides.

### 3.2 SEM analysis

Figure 2a–g demonstrates scanning electron micrographs of the studied treatments in two magnifications of 100× and 300×. According to our observations, the control group surface (Figure 2a) exhibited a glossy and homogenous pattern containing fat globules (Karami et al., 2008), while the CPE treatment image (Figure 2b) showed a nonglossy texture of the samples. Coating with CEO (Figure 2c) created a smoother and more uniform texture on the surface of the cheese compared to the control samples. The mixture of gelatin and starch particles (Figure 2d) created a nonporous and uniform layer with fluffy and velvety pattern on the surface of the cheese. Kaur et al. (2018) reported that the surface of wheat starch granules was found to be smoother than rice, maize, oats, sorghum, and millet starches. In agreement with their study, the wheat starch appeared to be spherical, lenticular, with large discs like granules on the cheese surface. The increase in gelatin concentration to 10% resulted in the aggregation of the granules and formation of beads on string structures (Torkamani et al., 2018). Adding CPE (Figure 2e) or CEO (Figure 2f) to GS coating led to the formation of cavities and pores in the surface; while, the combined addition of CPE and CEO to GS coating (Figure 2g) created no cavity and pore on the sample surfaces. In other words, GS‐CPE‐CEO treatment formed a firm, integrated, flawless, and homogenous layer on the cheese slice surfaces.

### 3.3 Microbiological analysis

**FIGURE 3:**
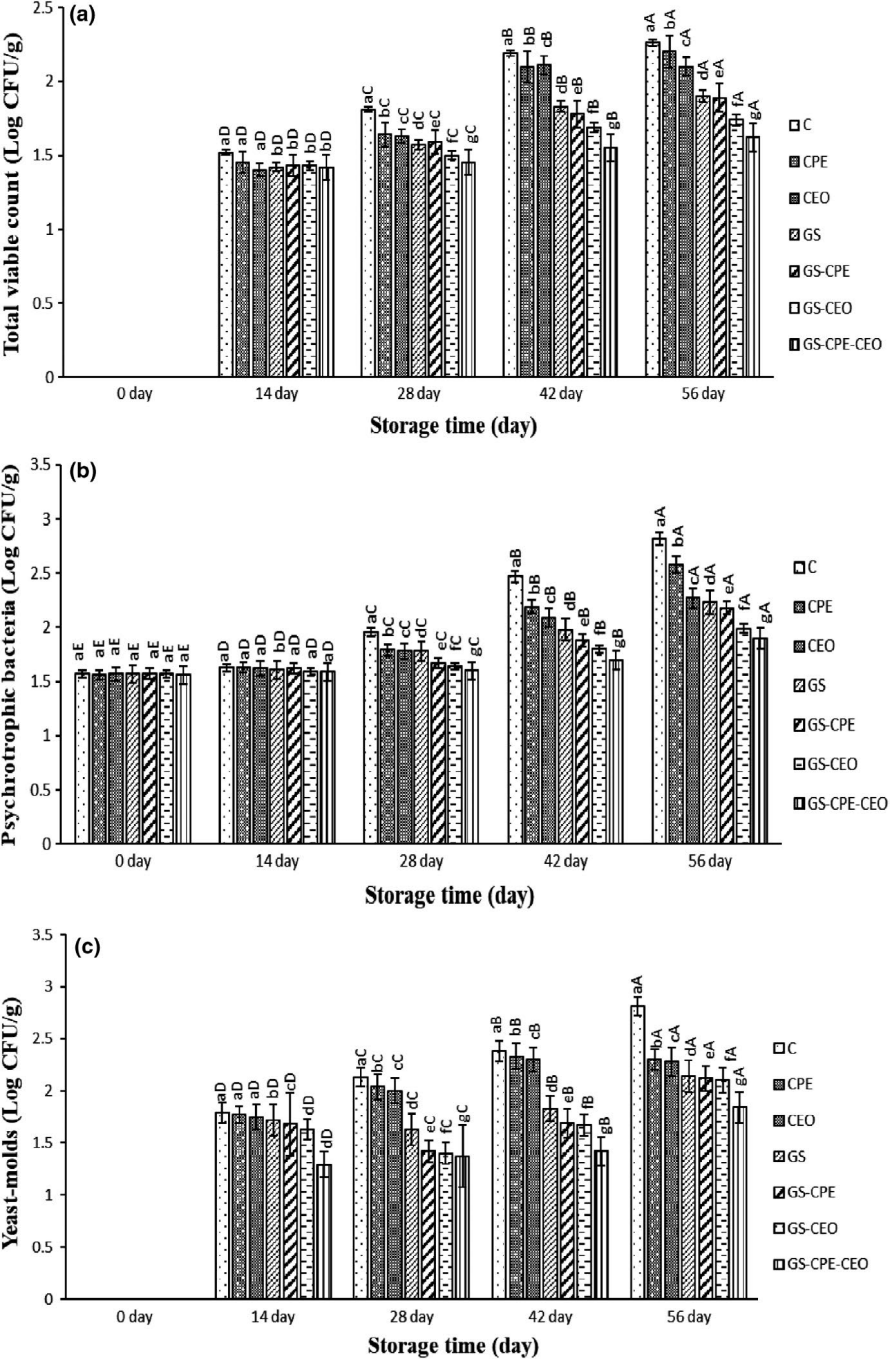
Average changes in total viable count (a), psychotropic bacteria (b), and yeasts–molds (c) of the cheese slices during storage at 4°C. Treatments: Sterile distilled water (C), cucumber peel extract (CPE), cumin essential oil (CEO), gelatin‐starch edible coating (GS), gelatin‐starch edible coating‐cucumber peel extract (GS‐CPE), gelatin‐starch edible coating‐cumin essential oil (GS‐CEO), and gelatin‐starch edible coating‐cucumber peel extract‐cumin essential oil (GS‐CPE‐CEO). Different letters within the same interval (day) (a, b, c, etc.) and the same treatment (A, B, C, etc.) indicate a statistically significant difference (*p* ≤ .05)

Figure 3a–c represents the total viable count (a), psychotropic bacteria (b), and yeast–molds (c) populations of the treated cheese during 56 days of storage. Total viable count and yeast–molds’ evaluation of cheese slices showed no detectable growth at day 0 of storage, while their growth increased after 14 days of storage and reached the range of 1.62–2.26 log CFU/g sample and 1.84–2.81 log CFU/g sample at the end of the storage period, respectively. The initial enumeration of psychotropic bacteria was in the range of 1.56–1.57 log CFU/g sample at day 0, reaching 1.90–2.82 log CFU/g sample on day 56. The observed pattern in all of the microbial groups of the studied samples was ascending until the end of the storage time. Similarly, in all of the studied microbial groups, the highest microbial count was found in the control group, followed by CPE, CEO, and GS. The GS‐CPE, GS‐CEO, and GS‐CPE‐CEO were in the next ranks, respectively. According to the results, GS containing CPE and CEO exhibited the strongest antimicrobial activities compared to the other treatments; so that the lowest microbial population (*p* ≤ .05) during the storage period belonged to the combined treatments, including GS‐CPE‐CEO, GS‐CEO, and GS‐CPE, respectively.

Moreno et al. (2018) concluded that gelatin:starch (1:1) films containing *N*‐α‐lauroyl‐l‐arginine ethyl ester monohydrochloride (LAE) notably elongated the shelf life of chicken breast meat during 20 days of storage. They reported that the activated gelatin:starch films by LAE significantly (*p* ≤ .05) decreased the total viable counts, psychotropic bacteria, lactic acid bacteria, anaerobic bacteria, total coliforms, and *Escherichia coli* of the refrigerated chicken breast meat compared to nonactivated ones during the storage period. A previous study found a significant decrease (*p* ≤ .05) in total mesophilic bacteria count of a regional cheese coated with a galactomannan‐based edible film that could be correlated to the low rate of oxygen transfer to the coated samples during the storage period (Cerqueira et al., 2010). Suppakul et al. (2008) reported that LDPE‐based films retarded the microbial growth (mesophilic bacteria and yeast–molds) in coated cheddar cheese during 25 days of storage at 4°C. Additionally, LDPE‐based films showed significant inhibitory activities against inoculated *L. innocua* and *E. coli* on Cheddar cheese during 40 and 16 days of storage period at 12°C (abuse condition) or 4°C in their study. In another study, the microbiological analysis of beeswax‐coated Kashar cheeses showed a decrease of 2.5 log units on yeast–mold counts compared to the uncoated samples at the 120th day of storage (Yilmaz & Dagdemir, 2012). Therefore, it can be concluded that the absence of oxygen can inhibit the growth of aerobic microorganisms on the product surface. In one research, the total viable count and psychotropic microorganisms of the chitosan–whey protein coated Ricotta cheese were significantly lower (*p* ≤ .05) compared to the uncoated samples over the storage time. The authors suggested a potential utility of chitosan–whey protein edible coating to extend the shelf life of Ricotta cheese (Di Pierro et al., 2011).

Derakhshan et al. (2008) showed that subjecting *Klebsiella pneumoniae* strains to subminimum inhibitory concentrations (sub‐MICs) of CEO resulted in cell elongation and repression of capsule expression and urease activity. They reported cumin aldehyde as the major ingredient of the CEO. Another study showed that the incorporation of CEO into a natural hydrocolloid of Shahri Balangu (*Lallemantia royleana*) seed mucilage significantly (*p* ≤ .05) enhanced the shelf life of refrigerated beef compared to the uncoated and not CEO‐containing samples. They observed the significant (*p* ≤ .05) and dose‐dependent antibacterial activity of CEO at different concentrations of 0.5%, 1%, 1.5%, and 2% (Alizadeh Behbahani et al., 2020). Abdellah et al. (2020) reported the in vitro antifungal activity of cumin seed essential oil and concluded that CEO can be considered as an effective treatment against *Candida albicans*.

### 3.4 Weight loss

**FIGURE 4:**
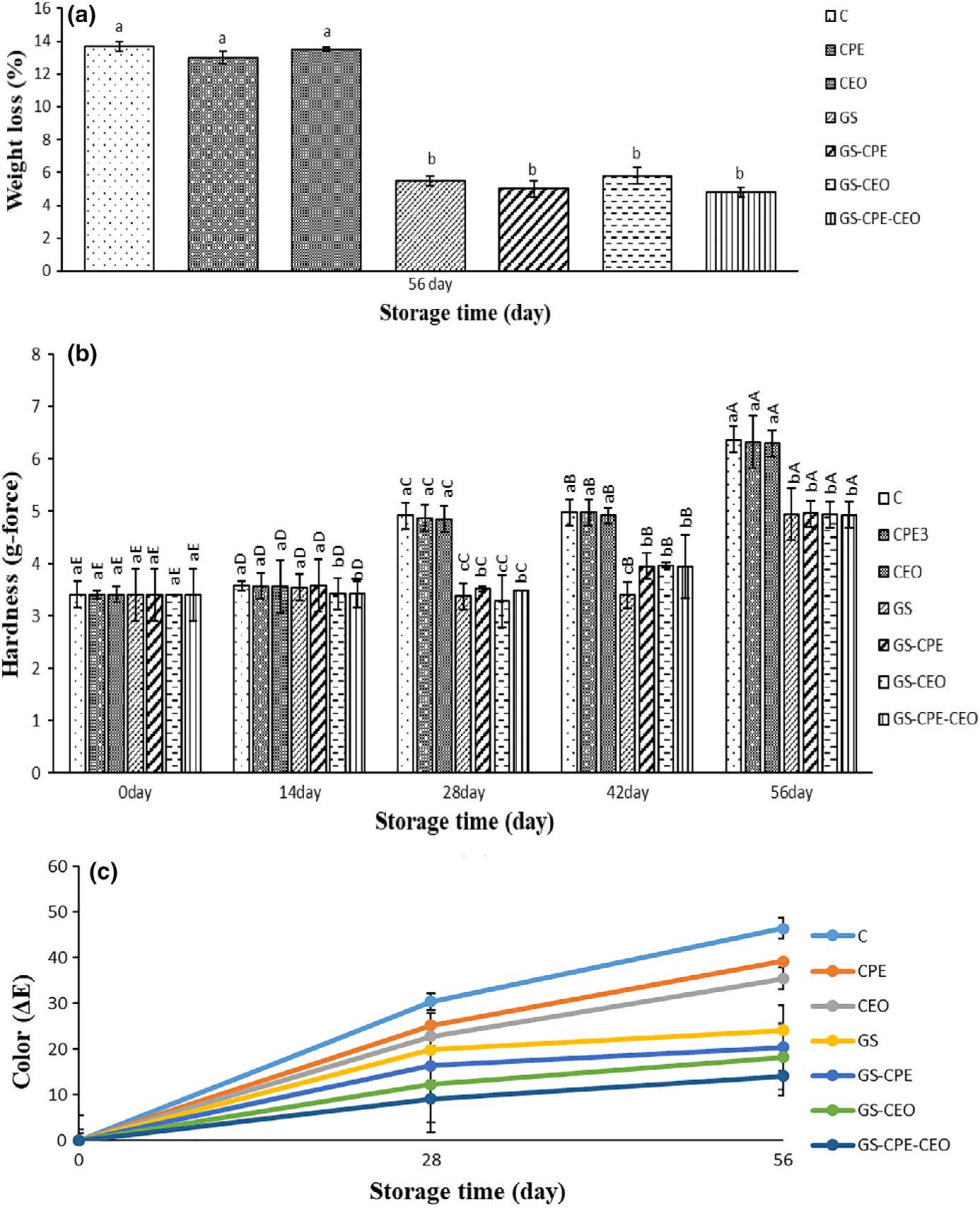
Average changes in weight loss (a), hardness (b), and color (c) of the cheese slices during storage at 4°C. The significance of the acronyms is the same as in Figure 3. Different letters within the same interval (day) (a, b, c, etc.) and the same treatment (A, B, C, etc.) indicate a statistically significant difference (*p* ≤ .05)

**FIGURE 5:**
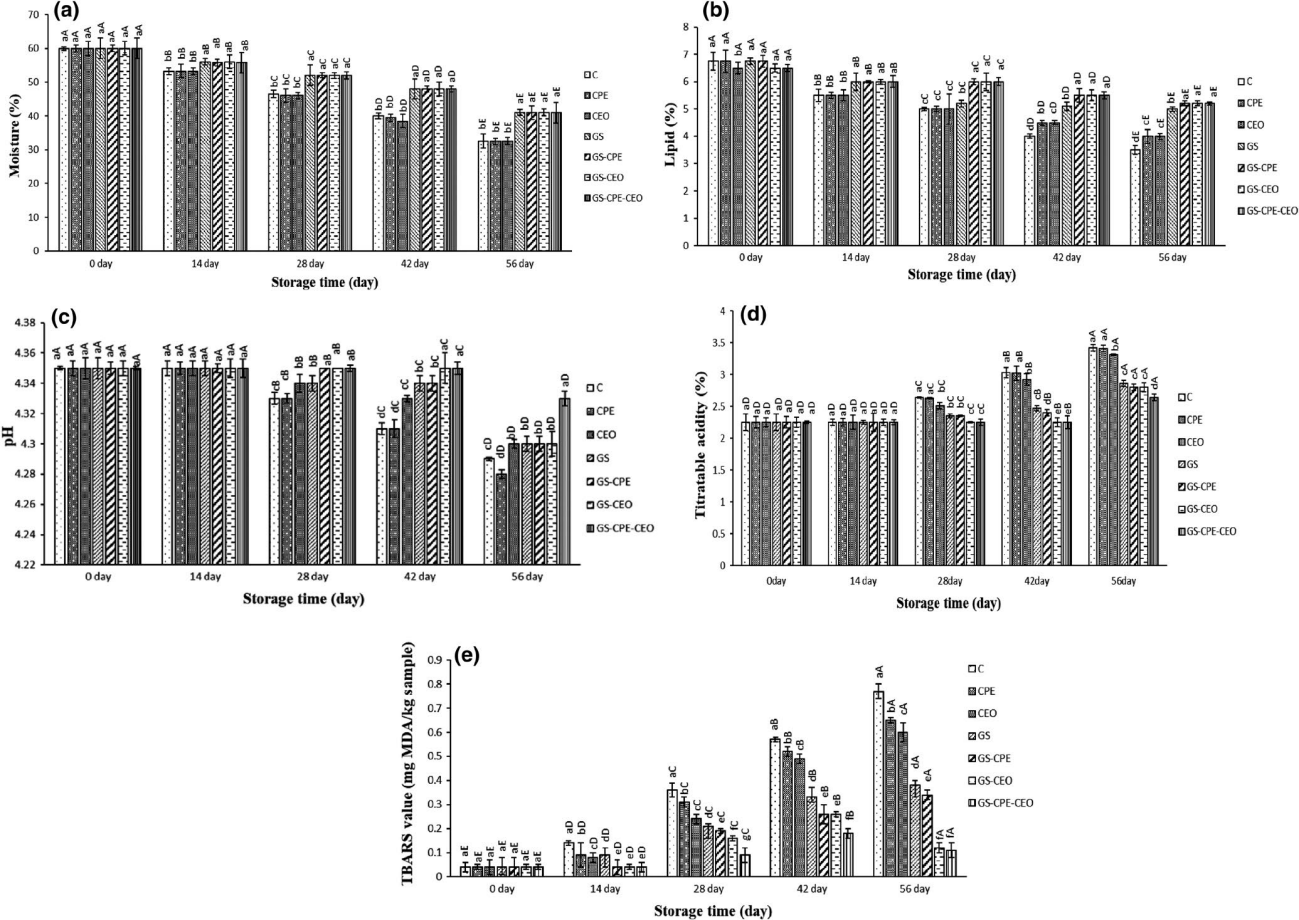
Average changes in moisture (a), lipid (b), pH (c), titratable acidity (TA) (d), and thiobarbituric acid reactive substances (TBARS) (e) of the cheese slices during storage at 4°C. The significance of the acronyms is the same as in Figure 3. Different letters within the same interval (day) (a, b, c, etc.) and the same treatment (A, B, C, etc.) indicate a statistically significant difference (*p* ≤ .05)

Weight loss is determined by the weight difference of initial weight (*W*_1_) of the samples and secondary (*W*_2_) weight of them during the storage period that can be correlated to the evaporation of water or volatile compounds, oxidation, and deformation of sample constituents such as lipid and protein during storage period. In other words, weight loss of cheese depends not only on moisture loss but also on cheese chemical reactions during storage period (Cipolat‐Gotet et al., 2020; Riahi et al., 2007). Figure 4a presents the changes in the weight of treated cheese slices during storage time. The GS edible coating significantly (*p* ≤ .05) decreased the weight loss of the coated samples compared to the uncoated ones at the end of storage time. According to the weight loss (Figure 4a), moisture (Figure 5a), lipid (Figure 5b), and TBARS (Figure 5e) results, the used GS coating could significantly (*p* ≤ .05) inhibit chemical reactions that cause weight loss in cheese slices compared to the uncoated ones during the storage period.

Generally, the biodegradable coatings can decrease weight loss (Figure 4a) in comparison to the uncoated cheese. This can be due to the water barrier feature of the edible coatings. Pena‐Serna et al. (2016) reported 9% weight loss in Zein‐coated Minas Padrao cheese during 56 days of the storage. The reported weight loss of coated Mozzarella cheese with chitosan, sodium alginate, and soy protein isolate (SPI) (Zhong et al., 2014), and coated “Saloio” cheese with whey protein isolate (WPI) (Ramos et al., 2012) or galactomannan (Cerqueira et al., 2010) was more than 15% during 20 days of storage. However, the weight loss of GS‐coated samples in the present study was 5% at the end of the storage period, which can be related to the lower gas (water vapor, oxygen, etc.) permeability of the GS coating in comparison to other edible coatings.

### 3.5 Hardness

Figure 4b illustrates the hardness feature of the studied samples during 56 days of storage. The coated cheese with GS showed less hardness changes compared to the control, CPE, and CEO treatments during study period. Compared to the coated ones, the hardness of three uncoated treatments significantly (*p* ≤ .05) increased from the 28th day to the end of the storage period. This can be due to the GS coating ability in the preservation of the initial moisture and tenderness of cheese slices during refrigerated storage. The hardness increase in the coated samples was estimated to be 30% at the end of storage time; while, in previous research, the hardness increase was reported to be 400% in Saloio cheese coated with WPI (Ramos et al., 2012) and galactomannan (Cerqueira et al., 2010). This value was over 350% in Mozzarella cheese coated with soy protein isolate (SPI) (Zhong et al., 2014). Pena‐Serna et al. (2016) estimated 14% hardness increase in Minas Padrao cheese coated with activated Zein after 14 days of refrigerated storage. In the present study, the better water barrier features of the GS edible coating reduced moisture loss and resulted in a softer and cohesive UF cheese compared to other protein and polysaccharide coatings reported in other studies.

### 3.6 Color

Color is an important factor in the acceptance of fresh products. Figure 4c illustrates the color change feature of the studied samples during cold storage. According to the obtained results, by increasing the storage period, C, CPE, and CEO‐coated samples, respectively, revealed a yellow rind that changed the surface color and texture of the cheese. Similarly, Pena‐Serna et al. (2016) reported the formation of a yellow rind in the unpackaged Minas Padrao cheese samples during 56 days of storage. In the present study, the coated cheese slices showed surface color change immediately after being coated, which can be related to the opaque milky color of the GS and natural green color of the CPE that created a greenish color on the surface of the slices. The GS‐CPE, GS‐CPE‐CEO, GS‐CEO, and GS‐coated cheese samples exhibited higher color change compared to the other samples at day 0 of the storage time. Unlike the uncoated samples, the initial color changes (because of the natural color of the GS and CPE) of the coated slices were almost stable until the end of the storage period. A similar yellowish color change of cheese samples immediately after coating with Zein has been reported (Pena‐Serna et al., 2016). The continuous color changes in the uncoated samples during storage time can be attributed to cheese oxidation and dehydration, which was inhibited in the coated cheeses due to lower oxygen and light permeability. The opacity and protective barrier properties of the used coatings led to producing less cheese rind (Cerqueira et al., 2009; Ramos et al., 2012).

### 3.7 Moisture

Figure 5a–g represents the alterations in chemical properties of the studied samples during 56 days of storage. The initial moisture of cheese slices was 60% at the first day of analysis (Figure 5a). By increasing the length of the storage period, the moisture content of all groups decreased. The coated samples, such as GS, GS‐CPE, GS‐CEO, and GS‐CPE‐CEO, significantly (*p* ≤ .05) showed less moisture loss (41%) compared to the uncoated ones, such as C, CPE, and CEO (32.5%), over the storage time. These results are in agreement with the weight loss values. Cerqueira et al. (2010) reported a high moisture loss in uncoated Regional cheese compared to the ones coated with galactomannan edible coating during 21 days of storage at 4°C and 25°C. In another study, the Zein edible coating decreased the moisture loss of Minas Padrao cheese in comparison to the uncoated ones (Pena‐Serna et al., 2016). Similarly, Ramos et al. (2012) observed lower moisture loss in the coated Saloio cheese with WPI coating compared to the uncoated samples during 60 days of storage at 10°C.

### 3.8 Ash and protein

According to the obtained results, the amounts of protein (12%) and ash (6%) of all treatments showed no changes and significant differences (*p* > .05) during the storage period. For this reason, these values were not presented in a graph or table in the present study; because, the protein and ash of all samples were 12% and 6%, respectively, and presented no changes during 56 days of storage. A previous study found no differences in ash and protein content of activated Zein‐coated and uncoated cheese during 56 days of refrigerated storage (Pena‐Serna et al., 2016). Although Guerra‐Martínez et al. (2012) reported a significant difference (*p* ≤ .05) in the protein content of Mexican Panela cheeses during 15 days of storage; but, the range of observed changes in protein content of the samples was very low, reported to be 19.39%–22%. Furthermore, they reported no obvious ascending or descending trend in the protein content of the studied samples throughout the storage time. In other words, they found an oscillating trend in the protein content changes of the studied cheeses during storage time.

### 3.9 Lipid

By increasing the storage period, the lipid content trends of the cheese slices were significantly (*p* ≤ .05) descending (Figure 5b). The lowest lipid content belonged to the control samples (3.5%), and CPE and CEO treatments (4%) were in the next ranks at the end of the storage time. The lipid content values of the combined coated cheese, such as GS‐CPE, GS‐CEO, and GS‐CPE‐CEO, were significantly (*p* ≤ .05) higher than those of the other slices during storage period. This was likely caused by the higher spoilage reactions, such as lipid oxidation and generation of volatile flavoring materials in the uncoated samples, compared to the coated cheese slices over the storage time. Pena‐Serna et al. (2016) reported a similar pattern in the lipid content of Minas Padrao cheese throughout storage. They observed that the Zein‐coated cheese slices exhibited a greasy surface and wrinkled appearance after 21 days of storage due to the lipid migration to cheese surface. The Mozzarella cheese coated with SPI displayed the same surface appearance after 7 days of storage (Zhong et al., 2014). In contrast to our findings, Ramos et al. (2012) found no statistically significant differences between fat content of coated and uncoated Saloio cheeses during storage time.

### 3.10 pH and TA

The initial pH of all samples (4.35) was stable until the 14th day of storage. Then, a descending pattern was observed until the end of the storage period, especially in the uncoated slices (Figure 5c). The lowest pH value belonged to the control (4.29), and others including CPE (4.28), CEO (4.30), GS (4.30), GS‐CPE (4.30), GS‐CEO (4.30), and GS‐CPE‐CEO (4.33) were in the next ranks, respectively. In agreement with pH values, TA of all samples showed an increasing pattern after 14 days of storage (Figure 5d). The initial TA value was 2.25% during 14 days and then it increased in all treatments until the end of the storage time. The lowest TA values (*p* ≤ .05) were found in GS‐coated samples compared to the uncoated ones. The pH loss of the studied samples can be related to the conversion of lactose to lactic acid and other volatile acids due to the activity of indigenous lactic acid bacteria. Ramos et al. (2012) reported a descending pattern in the pH values of all studied Saloio cheeses during 60 days of storage, although statistically significant differences (*p* ≤ .05) were not found between WPI and guar gum coated and uncoated samples. In another study, the low pH and high TA of beeswax‐coated and uncoated Kashar cheeses were linked to the accumulation of lactose metabolism products such as lactic acid and other volatile acids during the first 2 months of storage. After this time, a variable trend was observed in pH and TA values, which might be related to the alkaline components formed as a result of proteolytic degradation during the ripening period (Yilmaz & Dagdemir, 2012). In agreement with our findings, Di Pierro et al. (2011) showed a decreasing pattern in the pH values of chitosan/whey protein coated and uncoated Ricotta cheeses, after 7 days of storage. This was probably due to lactic acid synthesis by *Lactobacillus* spp., free fatty acids, and acidic amino acids’ production due to the lipolysis and proteolysis phenomena. In agreement with our results, they reported that TA of Ricotta cheese samples, especially the uncoated ones, increased during 30 days of storage.

### 3.11 Thiobarbituric acid reactive substances

The TBARS values of the studied cheese slices during cold storage are displayed in Figure 5e. By increasing the storage time, an ascending pattern in TBARS values of the cheese slices was found. The highest TBARS value (*p* ≤ .05) belonged to the control sample, and CPE, CEO, GS, GS‐CPE, GS‐CEO, and GS‐CPE‐CEO were in the next ranks, respectively. According to the Figure 4e, GS‐CPE‐CEO and GS‐CEO treatments showed the highest antioxidant activity (*p* ≤ .05) compared to the others during storage period. It seems that the combined use of GS coating along with CEO created the highest inhibition of oxidation reaction in cheese slices over the storage period. Unalan et al. (2013) reported that the Kashar cheese packed with Zein composite films containing lysozyme and antioxidant phenolic substances (catechin and gallic acid) showed significantly lower lipid oxidation than the uncoated samples during 35 days of cold storage. It has been reported that as a synthetic antioxidant, LDPE films containing BHT could enhance the oxidative stability of Asadero cheese during 100 days of storage (Soto‐Cantú et al., 2008).

### 3.12 Sensory analysis

Changes in the sensory characteristics (taste, odor, texture, and overall acceptability) of the cheese slices during storage period are presented in Table 1. There were no significant differences between all groups in terms of sensory characteristics until the 14th day. A descending pattern was observed in the sensory features of all treatments after 14 days until the end of the storage time. Based on the findings of the sensory analyses, the uncoated samples such as C, CPE, and CEO received the lowest scores by the panelists; while, the highest scores belonged to the coated cheese slices, including GS‐CPE‐CEO, GS‐CEO, GS‐CPE, and GS, respectively, after the 14th day until the end of the storage time. The combined treatments (GS‐CPE‐CEO, GS‐CEO, and GS‐CPE) showed more pleasant sensory features (*p* ≤ .05) compared to the others after the 14th day of storage. This can be due to the good preservation of GS coating along with the creation of attractive appearance by CPE and pleasant aroma and taste of CEO. Simultaneous use of the CPE and GS created an attractive greenish color on the sample surfaces. The texture score of the studied slices decreased over the storage time (*p* ≤ .05). The uncoated slices showed more decrease of texture scores (*p* ≤ .05) compared to the coated ones during refrigeration storage. These findings are consistent with the results of hardness measurements, which showed an increase in the loss of water during the storage period. Zhong et al. (2014) reported that the hardness of Mozzarella cheese increased dramatically with water evaporation. They observed that edible coatings generally delay the hardening process of cheese and produce the softer cheese texture compared to the uncoated ones, which may be attributed to their water retention ability. Guerra‐Martínez et al. (2012) reported that the hardness of fresh cheese has a high negative correlation with moisture levels (*R* = −.76); thus, a decrease in moisture levels during storage time results in hardness increase of the cheese. Cheese moisture itself showed to be an important factor defining cheese attributes, especially those regarding hardness; it seems that lower moisture loss of cheese during storage period may lead to the lower hardness of it. They reported that the hardness also has high negative correlations with protein content (*R* = −.83) and relative humidity (*R* = −.53). This is understandable, considering that the hydrolysis and hydration of casein protein contribute to the disintegration of casein matrix, as a result of which the hardness decreases. It is obvious that the moisture loss of cheese can decrease these reactions, leading to the hardness increase at the end.

**TABLE 1:** Changes in sensory attributes of the cheese slices during refrigerated storage

Storage period (days)	Treatments	Sensory attributes			
		Taste	Odor	Texture	Overall acceptability
28	C	4.5 ± 0.45^c^	4.1 ± 0.71^c^	4.2 ± 0.84^d^	4.4 ± 0.84^bc^
	CPE	4.3 ± 0.45^d^	4.2 ± 0.55^bc^	4.3 ± 0.45^c^	4.4 ± 0.45^bc^
	CEO	4.3 ± 0.45^d^	4.2 ± 0.55^bc^	4.3 ± 0.45^c^	4.4 ± 0.45^bc^
	GS	4.5 ± 0.45^c^	4.5 ± 0.45^b^	4.6 ± 0.45^b^	4.7 ± 0.45^b^
	GS‐CPE	4.5 ± 0.55^c^	4.7 ± 0.82^a^	4.7 ± 0.55^ab^	4.8 ± 0.71^a^
	GS‐CEO	4.6 ± 0.55^b^	4.7 ± 0.71^a^	4.8 ± 0.71^a^	4.8 ± 0.71^a^
	GS‐CPE‐CEO	4.7 ± 0.55^a^	4.8 ± 0.55^a^	4.8 ± 0.55^a^	4.8 ± 0.45^a^
42	C	4.3 ± 0.71^d^	4.1 ± 0.71^c^	3.1 ± 0.71^bc^	3.6 ± 0.71^c^
	CPE	4.2 ± 0.45^e^	4.1 ± 0.55^c^	3.2 ± 0.55^b^	3.7 ± 0.55^c^
	CEO	4.2 ± 0.45^e^	4.1 ± 0.55^c^	3.2 ± 0.55^b^	3.7 ± 0.55^c^
	GS	4.5 ± 0.55^c^	4.4 ± 0.55^b^	4.1 ± 0.45^ab^	4.2 ± 0.55^b^
	GS‐CPE	4.5 ± 0.55^c^	4.7 ± 0.82^ab^	4.1 ± 0.82^ab^	4.4 ± 0.82^b^
	GS‐CEO	4.6 ± 0.84^b^	4.8 ± 0.71^a^	4.1 ± 0.71^ab^	4.5 ± 0.82^a^
	GS‐CPE‐CEO	4.8 ± 0.84^a^	4.8 ± 0.55^a^	4.2 ± 0.55^a^	4.5 ± 0.82^a^
56	C	—	3.1 ± 0.45^d^	2.5 ± 0.45^b^	2.9 ± 0.45^e^
	CPE	—	3.4 ± 0.55^cd^	2.5 ± 0.55^b^	3.0 ± 0.45^d^
	CEO	—	3.5 ± 0.55^c^	2.5 ± 0.55^b^	3.0 ± 0.55^d^
	GS	3.5 ± 0.55^d^	4.5 ± 0.82^b^	3.5 ± 0.82^a^	4.0 ± 0.82^c^
	GS‐CPE	4.1 ± 0.45^c^	4.6 ± 0.82^ab^	3.5 ± 0.82^a^	4.2 ± 0.82^b^
	GS‐CEO	4.5 ± 0.82^b^	4.7 ± 0.55^a^	3.5 ± 0.55^a^	4.2 ± 0.55^b^
	GS‐CPE‐CEO	4.7 ± 0.71^a^	4.7 ± 0.55^a^	3.5 ± 0.55^a^	4.3 ± 0.55^a^

Note

The significance of the acronyms is the same as in Figure 3. Different letters within the same interval (day) (a, b, c, etc.) indicate a statistically significant difference (*p* ≤ .05).

Ramos et al. (2012) reported that uncoated regional Saloio cheese had a harder texture than the ones coated with WPI during the storage period. The researchers also reported similar sensory properties to our results in reducing odor scores in uncoated specimens. Yilmaz and Dagdemir (2012) showed that the highest overall acceptability score belonged to the coated Kashar cheese with beeswax compared to uncoated or vacuum‐packed samples during 120 days of storage.

## 4 CONCLUSION

The hydroethanolic CPE with MAE method showed more total phenolic content, reducing power, and DPPH and ABTS radical scavenging activities compared to the other extracts. The most efficient treatment in the shelf life elongation of the cheese slices was the GS‐CPE‐CEO group during 56 days of storage under refrigerated conditions. In addition, simultaneous usage of GS, CEO, and CPE created an integrated, flawless, and homogenous layer on the cheese slices and significantly postponed the physical, chemical, and microbial spoilage of the cheese samples during 56 days of storage. It is noteworthy that the designated formulation not only did not have inappropriate effects on the organoleptic features of the UF cheese slices but also made a palatable and pleasant feeling in the panelists. Therefore, the usage of GS coating containing CPE and CEO is proposed for improving the shelf life characteristics of UF cheese during 56 days of storage.

## CONFLICT OF INTEREST

The authors have declared that no conflicts of interest exist.

## AUTHOR CONTRIBUTIONS

**Zahra Esparvarini:** Conceptualization (equal); Data curation (equal); Formal analysis (equal); Investigation (equal); Software (equal); Writing – original draft (equal). **Behnaz Bazargani‐Gilani:** Conceptualization (equal); Formal analysis (equal); Investigation (equal); Methodology (equal); Software (equal); Supervision (equal); Validation (equal); Writing – review & editing (equal). **Mohammadreza Pajohi‐Alamoti:** Conceptualization (equal); Investigation (equal); Methodology (equal); Software (equal); Validation (equal); Writing – review & editing (equal). **Alireza Nourian:** Data curation (equal); Investigation (equal); Methodology (equal); Software (equal); Validation (equal); Writing – review & editing (equal).

## ETHICAL APPROVAL

This study does not involve any human or animal testing.

## Data Availability

Research data are not shared.
